# Can time-lapse culture combined with artificial intelligence improve ongoing pregnancy rates in fresh transfer cycles of single cleavage stage embryos?

**DOI:** 10.3389/fendo.2024.1449035

**Published:** 2024-08-29

**Authors:** Xiao Wang, Qipeng Wei, Weiyu Huang, Lanlan Yin, Tianzhong Ma

**Affiliations:** ^1^ Reproductive Medicine Center, Affiliated Hospital of Guangdong Medical University, Zhanjiang, Guangdong, China; ^2^ Department of Reproductive Medicine Center, Xiangyang Central Hospital, Affiliated Hospital of Hubei University of Arts and Science, Xiangyang, Hubei, China

**Keywords:** time-lapse culture, artificial intelligence, single cleavage stage embryo transfer, iDAscores, fresh cycle

## Abstract

**Purpose:**

With the rapid advancement of time-lapse culture and artificial intelligence (AI) technologies for embryo screening, pregnancy rates in assisted reproductive technology (ART) have significantly improved. However, clinical pregnancy rates in fresh cycles remain dependent on the number and type of embryos transferred. The selection of embryos with the highest implantation potential is critical for embryologists and influences transfer strategies in fertility centers. The superiority of AI over traditional morphological scoring for ranking cleavage-stage embryos based on their implantation potential remains controversial.

**Methods:**

This retrospective study analyzed 105 fresh embryo transfer cycles at the Centre for Reproductive Medicine from August 2023 to March 2024, following IVF/ICSI treatment at the cleavage stage. All embryos were cultured using time-lapse technology and scored using an automated AI model (iDAScore V2.0). Embryos were categorized into three groups based on the iDAScore V2.0: Group A (8 cells, iDA: 1.0-5.7); Group B (8 cells, iDA: 5.8-8.0); and Group C (>8 cells, iDA: 5.8-8.0). Clinical treatment outcomes, embryonic development, and pregnancy outcomes were analyzed and compared across the groups.

**Results:**

Baseline characteristics such as patient age, AMH levels, AFC, and basal sex hormones showed no significant differences among the three groups (p > 0.05). The iDAscores were significantly higher in Group C (7.3 ± 0.5) compared to Group B (6.7 ± 0.5) and the iDAscores were significantly higher in Group B (6.7 ± 0.5) compared to Group A (4.8 ± 1.0) (p < 0.001).

The mean number of high-quality embryos was highest in Group C (4.7 ± 3.0), followed by Group B (3.6 ± 1.7) and Group A (2.1 ± 1.2) (p < 0.001). There was no statistical difference (p = 0.392) in the ongoing pregnancy rate for single cleavage-stage transfers between Group B (54.5%, 30/55) and Group A (38.1%, 8/21), although there was a tendency for Group B to be higher.

**Conclusion:**

Combining time-lapse culture with AI scoring may enhance ongoing pregnancy rates in single cleavage-stage fresh transfer cycles.

## Introduction

Assisted Reproductive Technology (ART) has evolved significantly over the past 46 years and its effectiveness is widely recognized. To date, more than 10 million babies have been born through ART, effectively addressing the fertility issues of many infertile families (1). However, the live birth rate remains at 30-40%, indicating substantial room for improvement. Key factors for successful pregnancies include the *in vitro* culture of high-quality embryos, selection of embryos with the highest implantation potential, and synchronization with the optimal uterine implantation window. The rapid advancements in industrialization and artificial intelligence (AI) have introduced time-lapse culture, facilitating the *in vitro* culture and selection of embryos with superior implantation potential. While several studies have demonstrated that time-lapse culture can improve pregnancy rates, others have reported no significant impact on live birth rates (2–4).Specifically, using a time-lapse selection model to choose blastocysts for fresh single embryo transfer on Day 5 has not shown improvement in ongoing pregnancy rates compared to traditional morphology-based selection (5).

Despite the ongoing debate regarding the effectiveness of time-lapse culture, our study demonstrates that time-lapse culture combined with AI improves pregnancy rates in fresh cycles. Furthermore, single cleavage-stage transfers effectively reduce multiple birth rates and the risk of canceling fresh cycle blastocyst transfers. In developing countries, due to economic conditions and health insurance systems, physicians and patients may opt to transfer multiple embryos to achieve higher pregnancy rates. However, this practice increases the risk of multiple pregnancies, which in turn elevates the rates of miscarriages and perinatal complications for both mothers and infants, including preterm births and low-birth-weight babies (6, 7). Selective single embryo transfers are internationally recommended, primarily for blastocysts due to their high developmental potential. However, in fresh cycles, blastocyst transfer poses risks such as increased cancellation rates, reduced pregnancy rates due to the closure of the endometrial implantation window caused by certain ovulation regimens (e.g., antagonists), and an imbalance in the sex ratio (8). Single cleavage-stage transfers can effectively mitigate these issues. The current challenge is that traditional morphological assessment has limited predictive value for the developmental potential of cleavage-stage embryos, with implantation rates remaining around 20-40%. Ranking cleavage-stage embryos by their developmental potential is a significant challenge for embryologists. When only a single cleavage-stage embryo is transferred, the pressure on embryologists to make accurate selections increases, particularly when many high-quality embryos are available. Traditional morphological scoring can be subjective and inconsistent, leading to arbitrary selections (9). Current time-lapse culture technology, combined with AI-based selection systems, can rank embryos more effectively based on developmental dynamics, potentially outperforming traditional methods. This approach can shorten the time to first pregnancy, especially in fresh transfer cycles (10). This study is the first to analyze pregnancy outcomes based on different iDAScore groupings under the same morphological scoring of cleavage-stage embryos. This comparison allows for an evaluation of the validity of traditional morphological scoring versus AI-enhanced time-lapse culture scoring, providing data to support the broader application of time-lapse culture.

## Materials and methods

### Patients and study design

**Figure 1:**
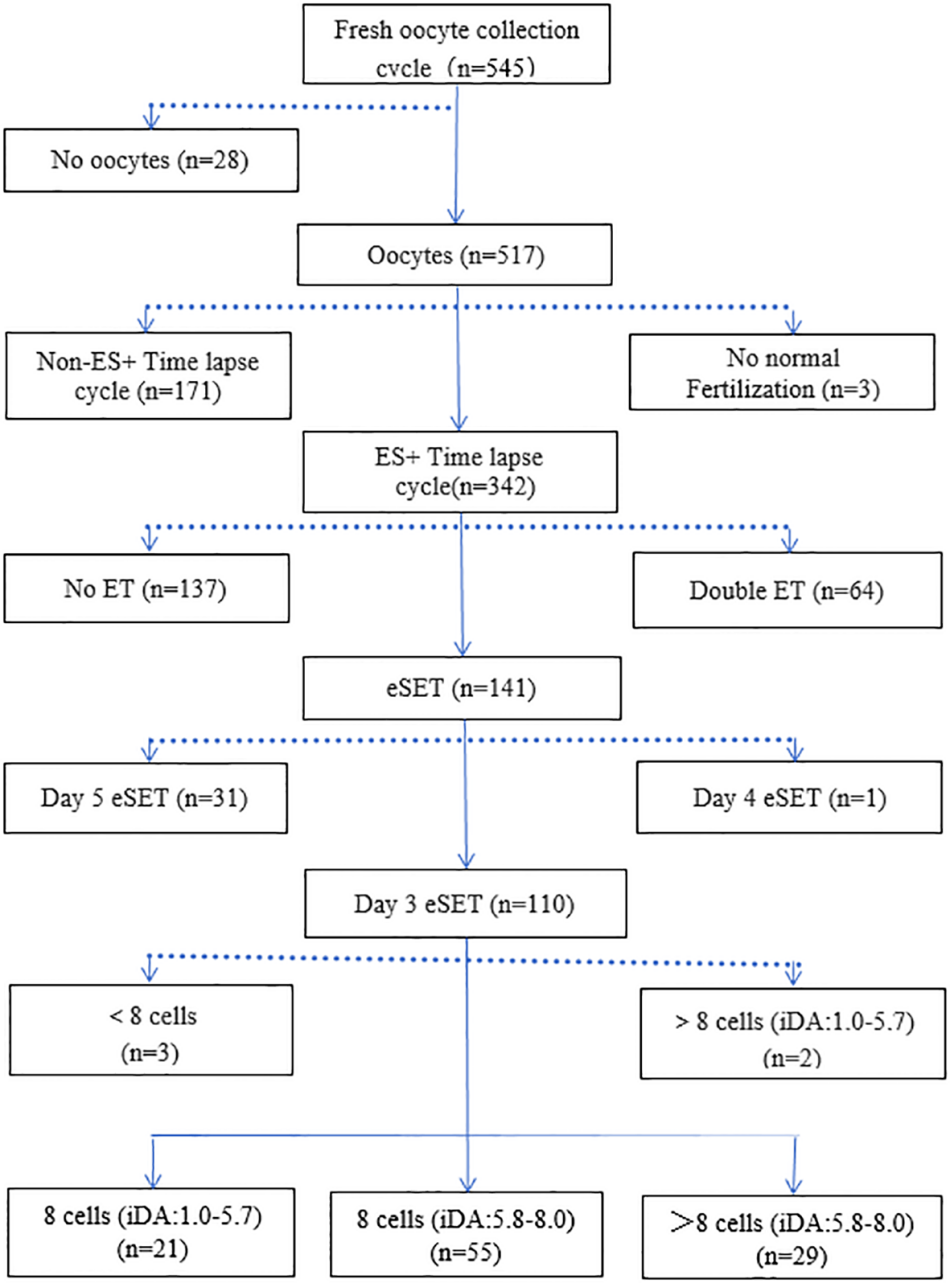
Flow chart of the study. All 545 fresh Oocytes retrieval cycles were left after inclusion exclusion criteria with 105 fresh cycles of single cleavage stage transplantation cycles.

This retrospective analysis included patients who underwent IVF/ICSI-assisted conception at the Reproductive Center of the Affiliated Hospital of Guangdong Medical University between August 1, 2023, and March 30, 2024. All procedures involving human participants adhered to the ethical standards of the institutional and national research committee, as well as the 1964 Helsinki Declaration and its subsequent amendments. Written informed consent was obtained from all patients, and they were informed that they could withdraw from the study at any time. Inclusion criteria for patients were: (i) age ≤38 years; (ii) long agonist protocols or antagonist protocols; (iii) fresh transfer on day 3 of a single cleavage-stage embryo cycle; (iv) embryos cultured in a time-lapse system (EmbryoScope+). Exclusion criteria included: (i) reproductive system abnormalities and chromosomal anomalies; (ii) a history of uterine surgery; (iii) missing data. The iDAScore values of the transferred embryos were evaluated using iDAScore Version 2.0. After applying the inclusion and exclusion criteria, 105 fresh cycles of single cleavage-stage embryo transfers were included from the initial 545 fresh oocyte retrieval cycles (**Figure 1**). The iDAScore is an automated AI model, and day 3 embryo morphological assessment was conducted based on the Istanbul consensus report, which serves as a conventional morphological grading method. Embryos were divided into three groups according to the iDAScore V2.0 score of 8 cells and >8 cells: Group A: 8 cells (iDA: 1.0-5.7); Group B: 8 cells (iDA: 5.8-8.0); Group C: >8 cells (iDA: 5.8-8.0).

### Controlled ovarian stimulation

Patients underwent controlled ovarian stimulation using either a long gonadotropin-releasing hormone (GnRH) agonist (afolin, Huiling, Germany) protocol or a GnRH antagonist (Cetrotide, Merck Serono, Germany) protocol, based on their ovarian response and medical history of ART treatment. Human chorionic gonadotropin (HCG, Zhuhai Lizhu, China; Ovidrel, Merck Serono, Germany) was administered when the diameter of at least one follicle reached 19 mm, two follicles reached 18 mm, or three follicles reached 17 mm. Additionally, the blood E2 level had to reach 250-300 pg/mL for each dominant follicle (≥ 16 mm), or more than 60% of the follicles greater than 16 mm. The injection of HCG was 5000-10,000 IU on the same night.

### Fertilization, embryo culture and transfer

IVF or intracytoplasmic sperm injection (ICSI) insemination was performed based on the patient’s condition. IVF was performed 38-40 hours after HCG administration at a concentration of approximately 100,000 spermatozoa/700 μL microdrop. Oocytes were denuded of cumulus cells 4-6 hours after IVF insemination to evaluate the extrusion of the second polar body. Oocytes showing a second polar body were transferred to the EmbryoScope+ for culture. Alternatively, oocyte denudation was performed 38-40 hours after HCG administration, and ICSI was conducted 2 hours later. After microinjection, all oocytes were individually cultured in the EmbryoScope+ time-lapse incubator under 6% CO_2_ and 5% O_2_ at 37°C. Embryo culture was carried out using continuous medium GTL culture medium (Vitrolife, Gothenburg, Sweden) until 66-68 hours after fertilization.Embryo grading refer to the Istanbul Consensus (11).

The iDAScore AI model, developed using deep learning and a neural network, was trained to analyze sequences of time-lapse images. The only inputs to the model were images from the time-lapse sequences, and the outputs were numerical scores from 1.0 to 8.0 (Day 3 Models) that correlated with the likelihood of an FHB. Therefore, iDAScore did not use any human-annotated data for training. The selection of embryos for transfer was based primarily on the iDAScore 2.0. Selected single embryos were incubated in G2 culture medium until the time of transfer.

A serum hCG level > 5 U/L was considered positive. Twenty-eight days after transplantation, a guided B ultrasound examination was performed to confirm the presence of an intrauterine pregnancy, and early cardiac motion indicated a clinical pregnancy.

### Statistical analysis

Data analysis was performed using SPSS 26.0 statistical software. The adoption rate (%) of enumeration data was used to indicate the comparison of rates between groups, using the χ^2^ test. Normally distributed data are expressed as mean ± SD, and the independent sample t-test was used for comparisons. The LSD t-test was used for pairwise multiple comparisons. A p-value < 0.05 was considered statistically significant.

## Results

### Participant characteristics

After applying the inclusion and exclusion criteria, a total of 105 fresh cycles of single cleavage-stage embryo transplantation were included in the analysis. Embryos with fewer than 8 cells rarely achieved the highest iDAScore, with only 3 fresh transfers in this category. Similarly, embryos with iDAScores of >8 cells in the 1.0-5.7 range had only two cases, which were excluded from the study due to the small sample size (**Figure 1**).

General data comparison focused on patients with different iDA scores of 8 cells and >8 cells. No statistically significant differences were found in baseline characteristics such as age, AMH, AFC, and basal sex hormones among the two groups of patients (p<0.05) (**Table 1**).

**Table 1:** Patients’ characteristics.

Characteristics	Group(A)	Group(B)	Group(C)	p-value
iDAScore v2.0 grouping	8 cells (iDA:1.0-5.7)	8 cells (iDA:5.8-8.0)	>8 cells (iDA:5.8-8.0)	
No. of cycles	21	55	29	/
Maternal age, (years)	31.4 ± 3.1	31.16 ± 3.5	32.8 ± 3.5	p=0.096
Body mass index (kg/m^2^)	22.1 ± 3.9	22.3 ± 2.7	23.0 ± 2.3	p=0.459
Infertility duration (years)	3.4 ± 2.7	3.0 ± 2.1	4.1 ± 3.0	p=0.174
Anti-mullerian hormone (AMH),(ng/mL)	3.1 ± 1.6	3.7 ± 2.5	4.4 ± 3.1	p=0.193
AFC, (num)	9.2 ± 4.6	11.1 ± 5.3	12.9 ± 7.5	p=0.099
Basic hormone				
Basal FSH(mIU/ml)	7.8 ± 2.8	6.8 ± 1.7	6.1 ± 1.6	p=0.056
Basal LH(mIU/ml)	6.8 ± 2.1	6.5 ± 3.8	7.3 ± 4.2	p=0.616
Basal PRL(ng/ml)	27.6 ± 20.3	30.5 ± 41.9	25.2 ± 23.6	p=0.811
Basal E_2_(pg/ml)	53.9 ± 34.7	45.6 ± 22.7	53.8 ± 54.8	p=0.515
Basal T(ng/ml)	0.3 ± 0.2	0.3 ± 0.1	0.3 ± 0.2	p=0.394
Basal P(ng/ml)	0.4 ± 0.2	0.4 ± 0.3	0.6 ± 1.0	p=0.356
Type of infertility				
Primary infertility(%),n/N	47.6(10/21)	41.8(23/55)	37.9(11/29)	p=0.791
Secondary infertility(%),n/N	52.4(11/21)	58.2(32/55)	62.19(18/29)	
Cause of infertility				
Tubal factor(%)	47.6(10/21)	38.2(21/55)	41.4(12/29)	p=0.535
Male factor(%)	33.3(7/21)	25.5(14/55)	10.3(3/29)	
Ovulation disorders(%)	9.5(2/21)	18.2(10/55)	17.2(5/29)	
Combination(%)	9.5(2/21)	7.3(4/55)	20.7(6/29)	
Endometriosis(%)	0(0/21)	5.5(3/55)	3.4(1/29)	
Unknown(%)	0(0/21)	3.6(2/55)	6.9(2/29)	
Ovarian hypoplasia(%)	0(0/21)	1.8(1/55)	00/29)	

Data are means ± SD or n.

### Stimulation cycle characteristics

The stimulation cycle was predominantly dominated by long agonist protocols (64.8%), while the antagonist protocol accounted for 35.2%. The difference in the percentage of protocols for ovulation promotion was not statistically significant in group A compared to group B, and group B compared to group C (**Table 2**).

**Table 2:** Stimulation cycle characteristics.

Characteristics	Group(A)	Group(B)	Group(C)	p-value
iDAScore v2.0 grouping	8 cells (iDA:1-5.7)	8 cells (iDA:5.8-8)	>8 cells (iDA:5.8-8)	
No. of cycles	21	55	29	
Ovulation programme				
Long agonist protocols(%)	76.2 (16/21)	63.6 (35/55)	58.6 (17/29)	p=0.425
Antagonist protocol(%)	23.8 (5/21)	36.4 (20/55)	41.4 (12/29)	
Initial Gn dose (IU)	160.7 ± 61.0	158.4 ± 53.8	146.6 ± 41.7	p=0.544
Length of Gn stimulation (days)	11.1 ± 1.6	10.7 ± 1.8	11.1 ± 2.1	p=0.615
Total dose of Gn (IU)	2172.0 ± 615.7	2024.6 ± 603.4	2052.1 ± 2701.2	p=0.661
E_2_ levels at the trigger day (pg/ml)	2633.2 ± 1359.5	2796.8 ± 1330.7	2465.37 ± 1088.9	p=0.538
LH levels at the trigger day (mIU/ml)	2.3 ± 0.9	2.7 ± 1.3	2.5 ± 1.5	p=0.422
P levels at the trigger day (mIU/ml)	0.6 ± 0.3	0.7 ± 0.3	0.7 ± 0.3	p=0.142
Endometrial thickness at the trigger day (mm)	12.5 ± 2.5	11.7 ± 2.2	11.3 ± 2.2	p=0.180

Data are means ± SD or n.

### Embryo laboratory data

The iDAScores were significantly higher in Group C (7.3 ± 0.5) compared to Group B (6.7 ± 0.5), and significantly higher in Group B compared to Group A (4.8 ± 1.0) (p < 0.001). However, there were no statistically significant differences between the groups in terms of fertilization program, number of oocytes, fertilization rate, oocyte cleavage rate, 4-cell rate, and 8-cell rate on DAY 2 and DAY 3.

The mean number of high-quality embryos was higher in Group B (3.6 ± 1.7) compared to Group A (2.1 ± 1.2) (p < 0.001). The number of blastocysts formed was higher in Group B (4.1 ± 2.2) than in Group A (2.5 ± 2.4) (p = 0.009). Additionally, the rate of blastocyst formation was higher in Group B (56.4 ± 28.4%) compared to Group A (37.9 ± 25.0%) (p = 0.008) (**Table 3**).

**Table 3:** Comparison of various fertilization programmes and embryo development data.

Characteristics	Group(A)	Group(B)	Group(C)	*p*-value
iDAScore v2.0 grouping	8 cells (iDA:1.0-5.7)	8 cells (iDA:5.8-8.0)	>8 cells (iDA:5.8-8.0)	
Cycle, n	21	55	29	
iDAScore v2.0	4.8 ± 1.0^a^	6.7 ± 0.5^b^	7.3 ± 0.5	*p*<0.001
Fertilization program				
IVF(%)	81.0(17/21)	69.1(38/55)	89.7(26/29)	*p*=0.104
ICSI(%)	19.0(4/21)	30.9(17/55)	10.3(3/29)	
Number of oocytes (n)	11.1 ± 5.2	12.31 ± 4.1	13.7 ± 6.0	*p*=0.183
MII (n)	9.4 ± 4.0	10.1 ± 3.5	12.0 ± 5.4	*p*=0.068
Number of normal fertilization (n)	6.8 ± 3.6	7.7 ± 2.5	8.7 ± 4.9	*p*=0.174
Normal Fertilization Rate (%)	62.0 ± 19.4	64.4 ± 18.3	65.1 ± 22.0	*p*=0.850
Number of cleavage (n)	8.0 ± 3.7	8.8 ± 2.8	9.6 ± 4.8	*p*=0.282
Oocyte cleavage rate (%)	97.8 ± 5.5	97.5 ± 8.7	98.2 ± 3.8	*p*=0.897
4-cell counts for Day2(n)	3.1 ± 1.3	4.3 ± 2.0	4.7 ± 3.5	*p*=0.065
4-cell rate for Day2(%)	52.9 ± 23.1	57.13 ± 20.6	53.64 ± 25.0	*p*=0.682
8-cell counts for Day3(n)	2.8 ± 1.5	3.5 ± 1.6	3.2 ± 2.6	*p*=0.331
8-cell rate for Day3(%)	49.3 ± 29.0	48.3 ± 19.0	37.8 ± 24.0	*p*=0.087
Number of good quality embryos on Day 3(n)	2.1 ± 1.2^a^	3.6 ± 1.7^b^	4.7 ± 3.0	*p*<0.001
Rate of good quality embryos on Day 3(%)	39.2 ± 25.8	50.0 ± 21.0	58.0 ± 27.0	*p*=0.025
Number of blastocysts formed(n)	2.5 ± 2.4^a^	4.1 ± 2.2	5.1 ± 4.0	*p*=0.009
Rate of blastocyst formation (%)	37.9 ± 25.0^a^	56.4 ± 28.4	63.2 ± 30.5	*p*=0.008

Data are means ± SD or n.

^a^compared with group B (p<0.05); ^b^compared with group C (p<0.05).

### Clinical outcomes

There was no statistically significant difference (p = 0.392) in the ongoing pregnancy rate for single cleavage-stage transfers between Group B (54.5%, 30/55) and Group A (38.1%, 8/21), although there was a tendency for the rate to be higher in Group B (**Table 4**).

**Table 4:** Comparison of clinical pregnancy, early abortion and ongoing pregnancy between various.

Characteristics	Group(A)	Group(B)	Group(C)	*p*-value
iDAScore v2.0 grouping	8 cells (iDA:1.0-5.7)	8 cells (iDA:5.8-8.0)	>8 cells (iDA:5.8-8.0)	
No. of cycles	21	55	29	
Clinical pregnancy (%)	47.6(10/21)	60.0(33/55)	69.0(20/29)	*p*=0.315
Early abortion (%)	20.0(2/10)	10.0(3/33)	20.0(4/20)	*p*=0.466
Ongoing pregnancy(%)	38.1(8/21)	54.5(30/55)	55.2(16/29)	*p*=0.392

### IDAscores predict the developmental potential of 8-cell cleavage stage embryos

**Figure 2:**
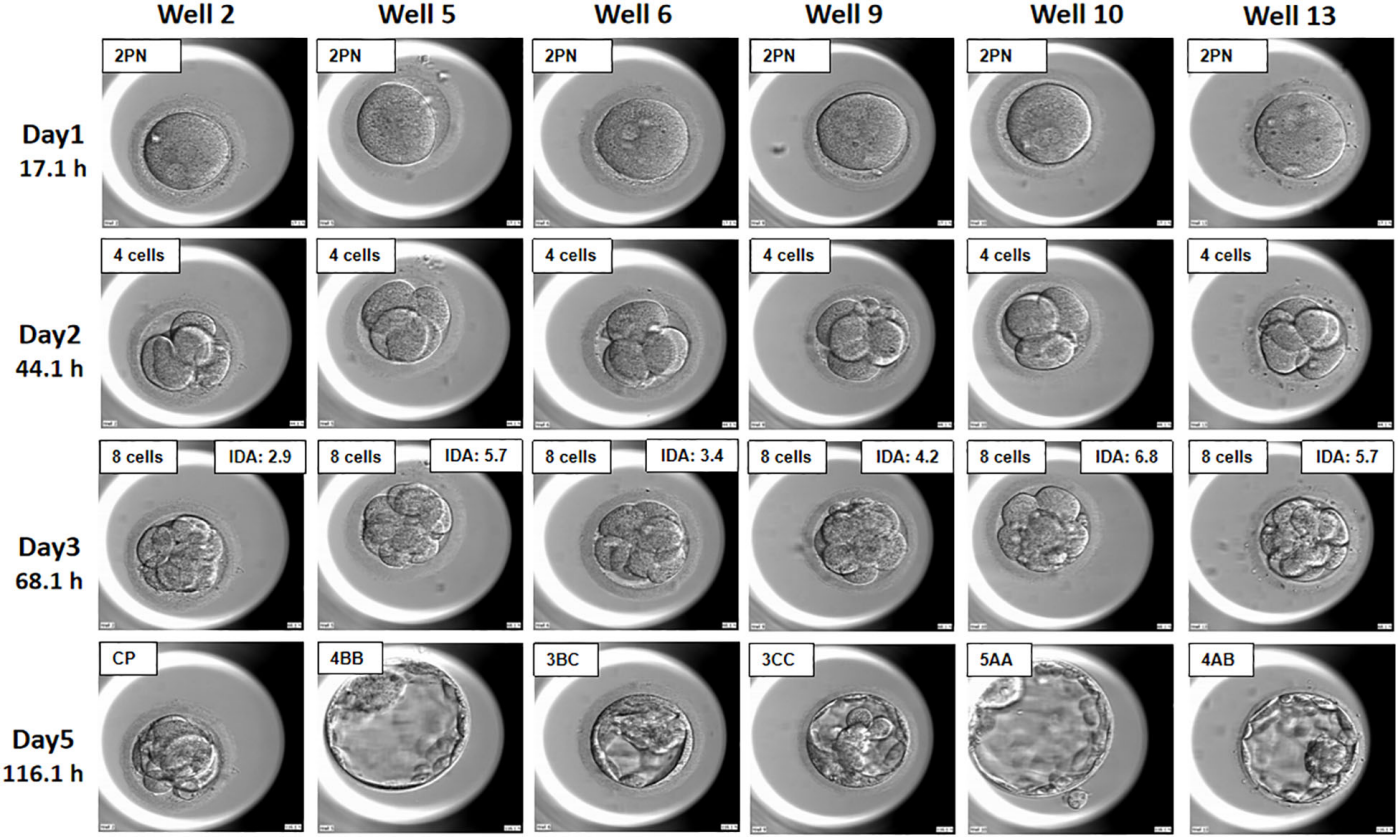
IDAscores predict the developmental potential of 8-cell cleavage stage embryos. Well 2, Well 5, Well 6, Well 9, Well 10and Well 13 were all rated as (8 cells) high-quality embryos by conventional morphology scores at 68.1 hours after fertilization (DAY3), but at 116.1 hours after fertilization (DAY5), only embryos from Well 5, Well 13 with an IDA score of 5.7, and Well 10, with an IDAscore of 6.8, developed into high-quality blastocysts in Day 5.

**Figure 2** illustrates embryos assessed by embryologists at the 8-cell level with varying developmental potentials. IDAscores proved to be reliable predictors of the probability of embryo development into blastocysts. For instance, embryos with iDAscores above 5.7, such as Well 10 (iDA=6.8), Well 5 (iDA=5.7), and Well 13 (iDA=5.7), developed into good-quality blastocysts. In contrast, embryos with iDAscores below 5.7, such as Well 2, Well 6, and Well 9, did not form usable blastocysts by Day 5. Additionally, **Supplementary Video 1** showcases the embryo developmental kinetics from 6.8 hours to 117.2 hours post-fertilization, highlighting variations in blastocyst formation despite similar Day 3 embryo quality assessments.

## Discussion

ART has evolved significantly over the years, leading to the emergence of various new technologies and techniques, such as advanced culture fluids (12, 13), heavy oils for assisted reproduction cultures (14), non-invasive preimplantation genetic testing (niPGT) (15), Raman spectroscopy applications (16, 17), time-lapse incubators (3, 18), and artificial intelligence applications (19, 20). Clinicians, embryologists, and scientists are continually striving to improve pregnancy rates, which are now approaching a plateau. The ultimate goal for every reproductive medicine practitioner is to select embryos non-invasively, economically, objectively, and conveniently, and implant them into the patient’s uterus to achieve pregnancy and successfully deliver a healthy baby (21). Literature has shown that both fresh cycle transfers and freeze-thaw cycle transfers result in good pregnancy rates when ovarian hyperstimulation syndrome (OHSS) is well controlled, but fresh cycle transfers effectively shorten the patient’s waiting time for implantation (22). Time-lapse culture combined with artificial intelligence to assess embryo kinetic parameters may serve as a non-invasive, effective, and concise method to enhance clinical pregnancy rates in fresh cycles. Ma et al. reported a correlation between 3,448 blastocyst biopsies and iDAscores, finding that time-lapse culture combined with iDAscores predicted embryonic viability, with a higher probability of viable blastocysts associated with higher iDAscores (23).

The choice between Day 3 cleavage stage embryo transfer and Day 5 blastocyst stage transfer in fresh cycles, and whether to transfer one or two embryos, are critical questions for clinicians and embryologists. Fresh transfer of blastocysts increases the implantation rate due to the selection of viable embryos through extended *in vitro* culture but also raises the rate of preterm birth (24). Some ovulation protocols, such as antagonist protocols, may miss the implantation window due to early closure of endometrial receptivity (25, 26), reducing the pregnancy rate of blastocyst transfer. The potential long-term effects of blastocyst culture, such as shortened telomeres and senescent phenotypes in offspring, should also be consideredt (27). Fresh blastocyst transfers also risk cancellation due to failure to develop or lack of transferable blastocysts on Day 5. Additionally, sex bias due to blastocyst transfer is a significant factor, with stricter regulations in some countries to avoid severe sex ratio imbalances (8).

The current 2024 updated ESHRE guideline on the number of embryos to transfer during IVF/ICSI recommends that selective single embryo transfers be performed up to the age of 38 years (moderate quality evidence), and also for women aged 38 years and above (very low quality evidence) (28). Transferring two or more cleavage stage embryos in fresh cycles has been the dominant strategy in developing countries due to the limited predictive value of traditional morphological assessments of implantation potential. This approach aims to guarantee pregnancy rates in a single transfer, despite the increased risk of multiple pregnancies and associated maternal and perinatal complications, such as gestational diabetes, pre-eclampsia, preterm labor, and low birth weight (29, 30).

Currently, traditional morphological evaluation can effectively distinguish between good quality and poor-quality embryos. However, for patients with a high number of good quality embryos, there is confusion in embryo sorting, making it difficult to select the best embryos. Different embryologists may select different embryos, which introduces subjectivity and arbitrariness, thus there is no guarantee that the transferred embryos are the most promising for single cleavage stage embryo transfer (20). Time-lapse culture combined with artificial intelligence screening can score and rank cleavage stage embryos based on their kinetic parameters, accurately predicting their developmental potential. Transferring Day 3 highly scored single cleavage stage embryos facilitates embryo and endometrium interactions, effectively reduces *in vitro* culture time, and ensures the pregnancy rate while minimizing the risks associated with blastocyst transfer. In this study, we found that although all embryos were morphologically assessed as good quality 8-cell embryos, their developmental potential and ongoing pregnancy rates varied according to their iDAscores (**Figure 2**). Embryos with higher iDAscores had higher ongoing pregnancy rates, though the difference was not statistically significant, likely due to the small sample size. Another key finding was that Day 3 embryos with both high cell counts and high iDAscores exhibited higher ongoing pregnancy rates. While the previous Istanbul consensus indicated that neither too slow nor too fast embryo development is optimal (11), our study found that Day 3 embryos with higher cell counts and iDAscores had the highest implantation rates. More data is needed to validate this phenomenon.

Time-lapse culture combined with iDAscores scoring also predicts the developmental potential of each embryo (31), allowing embryologists to accurately decide on strategies for transferring, freezing, and continuing to culture embryos based on the patient’s overall embryo score. If the patient has embryos with high iDAscores, a single cleavage stage embryo transfer is chosen. If the patient has low iDAscores, the number of embryos to be transferred can be determined by considering the patient’s individual situation, height, and uterine condition.

This personalized approach can simultaneously ensure the pregnancy rate and effectively reduce the number of embryos transferred, thereby reducing the rate of multiple births. This is important for stabilizing the pregnancy rate of a reproductive center and for maintaining the center’s reputation. Currently, due to the high cost of time-lapse incubators and special petri dishes, full time-lapse incubation may not be feasible in many fertility centers in developing countries.The limited number of time-lapse incubators can be prioritized for patients considering fresh cycle transfers to ensure pregnancy rates. For other patients unable to undergo a fresh transfer, the pregnancy rate can be maintained by transferring blastocysts in a freeze-thaw cycle, as the blastocyst culture effectively eliminates embryos with low developmental potential, thus improving the implantation rate.

The potential benefits of time-lapse culture with artificial intelligence have been added.The time-lapse culture generate a large amount of embryo image data that records a large number of kinetic parameters of embryo development. If these large amounts of data need to be labeled by embryologists, then this seriously affects the efficiency of embryologists in evaluating embryos, and also prevents the evaluation of effective parameter weights. And the time-lapse culture combined with artificial intelligence can effectively solve this problem. It can reduce the workload of embryologists and assist embryologists in selecting embryos with the highest developmental potential.

In summary, the strategy of time-lapse culture combined with AI for fresh cycle single cleavage stage embryo transfer is a non-invasive, rapid method that helps resolve transfer sequencing confusion for embryologists evaluating patients with multiple embryos. Combining time-lapse culture with AI scoring may enhance ongoing pregnancy rates in single cleavage-stage fresh transfer cycles.

## Data Availability

The original contributions presented in the study are included in the article/**Supplementary Materials**, further inquiries can be directed to the corresponding author/s.
